# Feasibility Analysis of CareToy-Revised Early Intervention in Infants at High Risk for Cerebral Palsy

**DOI:** 10.3389/fneur.2020.601137

**Published:** 2020-12-16

**Authors:** Elena Beani, Valentina Menici, Alessandra Cecchi, Maria Luce Cioni, Matteo Giampietri, Riccardo Rizzi, Giuseppina Sgandurra, Giovanni Cioni, CareToy-R Consortium, Claudia Artese

**Affiliations:** ^1^Department of Developmental Neuroscience, Istituto di Ricovero e Cura a Carattere Scientifico (IRCCS) Fondazione Stella Maris, Pisa, Italy; ^2^Division of Neonatology, Careggi University Hospital, University of Florence, Florence, Italy; ^3^Neonatal Intensive Care Unit, Children's Hospital A. Meyer, Florence, Italy; ^4^Neonatal Intensive Care Unit, Pisa University Hospital Santa Chiara, Pisa, Italy; ^5^Tuscan Ph.D. Programme of Neuroscience, University of Florence, Florence, Italy; ^6^Department of Clinical and Experimental Medicine, University of Pisa, Pisa, Italy

**Keywords:** early intervention, tele-rehabilitation, CareToy, information and communication technologies, infants, cerebral palsy

## Abstract

Infants with perinatal brain injury are at high risk for Cerebral Palsy (CP). Progresses in detection of early signs of brain injury and of CP allow early intervention (EI) programs for improving the outcome of these infants. CareToy system (CT), developed within a European project (Trial Registration: NCT01990183), allows providing, by means of tele-rehabilitation, a highly personalized, family-centered, home-based EI for young infants, remotely managed by clinicians. CareToy, already used with pre-terms without brain injury, has been adapted for high-risk infants in a project funded by the Italian Ministry of Health, and the CareToy-Revised (CareToy-R) has been realized (Trial registration: NCT03211533 and NCT03234959). Before assessing its efficacy, it was crucial to evaluate the acceptability, usability, and feasibility of CareToy-R EI. Nineteen high-risk infants with perinatal brain injury, aged 5.95 ± 2.13 months (range 3.12–10.78 months), carried out an 8-week training with CareToy-R at home, performing customized playful activities with their parents, tailored to their rehabilitative needs, remotely managed by clinicians. The feasibility of training and study procedures was assessed through criteria derived from literature; acceptability and usability have been analyzed from data about individual training and an *ad hoc* questionnaire. All CareToy-R trainings were planned by the clinical staff with a daily personalized use for each infant between 30 and 45 min (mean 34.37 min). The amount of executed training by the infants was very high (daily mean 30.30 min), with no differences related to infant age, sex, and gestational age. All the nine feasibility criteria were achieved, family compliance to the project was very good, data collection was completed and the CareToy-R system worked properly and easily for parents. The answers to the questionnaire had a total mean score of 84.49% and they ranged from a minimum of 81.05% (in “easy to use” area) to a maximum of 86.49% (“changes due to the training” area), with no differences related to nationality or familiarity with technology of the mothers. This study reports preliminary evidence to the feasibility of a home-based EI with CareToy-R system in infants at high risk for CP. Results of the RCT will provide data about the potential effectiveness of this approach.

## Introduction

Perinatal brain injury exposes infants to a high risk for developing cerebral palsy (CP), the most common cause of physical disability during the developmental age (1). Despite recent evidence of a genetic contribution to the pathogenesis of CP, the presence of an early brain injury still represents the most important causative factor (2).

Typical care pathways for infants born preterm or with congenital brain injury consists of dedicated neuroradiological and clinical follow-up programs which can be established if the infant is at high risk for developing a CP (3). Indeed, the combined predictive power of magnetic resonance imaging (MRI) and clinical assessment tools such as the General Movement Assessment (GMA) according to Prechtl or the Hammersmith Infant Neonatal Examination (HINE) allow establishing a diagnosis of CP as early as 3–5 months with high sensitivity and specificity (3–5). An early and accurate diagnosis of CP is crucial as it allows a prompt and individualized access to a rehabilitative intervention program in a critical period for brain development; this promptness allows maximizing the effectiveness of intervention, exploiting a window of maximal plasticity for many different developmental domains (motor, visual, cognitive…) (6).

In order to be maximally effective, an early intervention program (EI) should be intensive, personalized, family-centered, and affordable both for families and health services. Moreover, EI should include multi-axial activities targeting motor, cognitive, sensory, and social functions in an integrated systemic approach (7).

The recent availability of tele-rehabilitation tools has allowed the application of this rehabilitative approach to the home setting which is the most enriching and ecological environment for the infants (8). Moreover, the possibility of standardizing a methodology of intervention and of remotely acquiring quantitative measure during the EI program, thanks to biomechatronic toys and telemonitored systems, has created a promising opportunity for developing innovative EI programs. The home-based concept and the tele-rehabilitation architecture provide significant added value to EI programs and both represent the pillars of the CareToy (CT) system.

The CareToy system was created in the framework of a multicentric international project (www.caretoy.eu, Trial Registration: NCT01990183). It is a biomechatronic baby gym equipped with different types of sensors designed to provide a highly personalized, family-centered, home-based intervention for young infants, remotely managed by dedicated clinical and rehabilitation staff. CareToy has been validated in an RCT study involving a sample of Italian and Danish preterm infants at low risk for cerebral palsy (9): results showed an improvement of the visual and motor development, as well as a maternal reduction of levels of stress (10). In a feasibility study, an EI program using CareToy has been carried out in a small population of infants with Down Syndrome showing the good adaptability of the system to different populations (11).

Basing on this experience, an EI program using a revised version of CareToy system (CareToy-Revised, CT-R) has been implemented in an ongoing RCT involving high-risk infants with perinatal brain injury (Trial registration: NCT03211533 and NCT03234959) (12).

Before analyzing the clinical efficacy of the CareToy-R system on this population, the present study aims to investigate the rate of acceptability, usability, and feasibility of an EI based on CareToy-R in families with infants at high risk for cerebral palsy.

## Materials and Methods

This feasibility study is focused on the upgraded version of CareToy, the biomechatronic smart baby gym, that is, the CareToy-R, designed and adapted for infants with perinatal brain injury, at high risk for developing CP.

The study protocol of the RCT project and the detailed description of the system have been published elsewhere (12). The wide CareToy-R RCT study is a multi-center, paired, and evaluator-blinded study with two investigative arms of two EI: Infant Massage and CareToy-R training for a duration of 8 weeks, in which eligible infants are allocated randomly at baseline (T0). Details are shown above.

The study has been approved by Tuscany Pediatric Ethics Committee (84/2017), and it was registered (NCT03234959) on Clinical Trials.gov.

Before comparing the effects of the Infant Massage and CareToy-R training, the feasibility, acceptability, and usability of the CareToy-R system need to be investigated.

### Participants

Families of the subjects involved in this study were approached during the hospitalization in the Neonatal Intensive Care Units or during the neurodevelopmental follow-up programs in three different University Hospitals: the “Santa Chiara Hospital” in Pisa, the “Meyer Children's Hospital” and the “Careggi General Hospital,” both in Florence.

For the CareToy-R study, infants with any sign of perinatal brain injury upon neonatal brain ultrasonography (US) or magnetic resonance imaging (MRI) were considered eligible. After 3 months corrected age, all the infants were checked for the presence of atypical clinical signs (absence of physiological fidgety movements or abnormal score at the Hammersmith Infant Neurological Examination, HINE). The presence of both the clinical and the radiological criteria was considered mandatory in order to establish the high risk of CP and to offer the participation to the trial. The presence of polymalformative syndromes, cerebral malformations, severe sensory impairments (retinopathy of prematurity grade >II, deafness, or blindness) was considered exclusion criteria.

Parents or legal representatives signed the informed consent forms to accept the inclusion of the infant in the study. Recruitment for this preliminary study on feasibility started after the approval of the research project by the Ethics Committee. Intervention could start when the infant reached some pre-established motor skills (starting from the initial head control), which are expressed by the cut-off score of gross motor area of the Ages & Stages Questionnaire (last additional criterion).

For this feasibility study, eligible families were those of infants randomized to the CareToy-R arm of the project.

### Study Design and Procedures

The recruitment of the whole CareToy-R project has started on September 2017 and has been completed on June 2020.

The minimum sample size required was 19 for the CareToy-R group.

After the enrolment, infants were allocated to one of the two investigative arms: CareToy-R training or Infant Massage [for details see Sgandurra et al. (12)]. Both interventions lasted 8 weeks and during this phase infants continue to receive standard care provided by National Health System.

Assessments, already described in the study protocol (12), were performed by a child neurologist and a therapist at the following times:

- T0, the baseline, in the week before CareToy-R training or Infant Massage
- T1, the primary endpoint, within a week after the end of the training
- T2, after 8 weeks after the end of the training
- T3, last follow-up, at 18 months of (corrected) age of the infant.

The Infant Motor Profile [IMP, (13, 14)], primary outcome measure of the RCT CareToy-R study, is a video-based assessment of motor behavior of infants. Peabody Developmental Motor Scales—Second Edition [PDMS-2, (15, 16)], Bayley Scales of Infant Development [BSID-III, (17)] Cognitive subscale, standardized video-recordings of parent-infant interaction (18, 19), Teller Acuity Cards® (20), and Actigraphic analysis [Motionlogger Microwatch, (21)] were included as secondary measures. In addition, two questionnaires were administered to parents at all assessment times: BSID-III social-emotional scale (22) and Parenting stress index [PSI, (23)].

These assessment tools were administered at all assessment times.

Finally, families allocated to the Caretoy-R arm of the study were asked to fill in two questionnaires: the familiarity with technology (24) and “CareToy-Revised Questionnaire Parent-Infant Experiences” (see details below).

This last questionnaire, related to the feasibility aim, will be the measure reported in this study.

### Intervention

#### CareToy-Revised System

CareToy is a technological smart system equipped with sensorized toys, developed as a telerehabilitation tool for home EI.

As described in literature (25, 26), it is a biomechatronic gym, composed by: (i) two feedback walls containing button, wires for toys, lights and speakers; (ii) a wall with a screen where specially developed pictures and videos are shown; (iii) a wall for the positioning of sitting posture modules; (iv) sensorized toys with different shapes; (v) an arch with lights and wires for toys; (vi) videocameras for recording infant's behavior; (vii) a sensorized mat; (ix) a kit of wearable sensors; and (x) a laptop with an *ad-hoc* software.

As described in the study design paper (12), in order to be adapted to the new population of infants, while maintaining the flexibility and variability of the proposals, the clinical staff planned to make some small but essential modifications to the CareToy system, creating the CareToy-R system.

**Figure 1:**
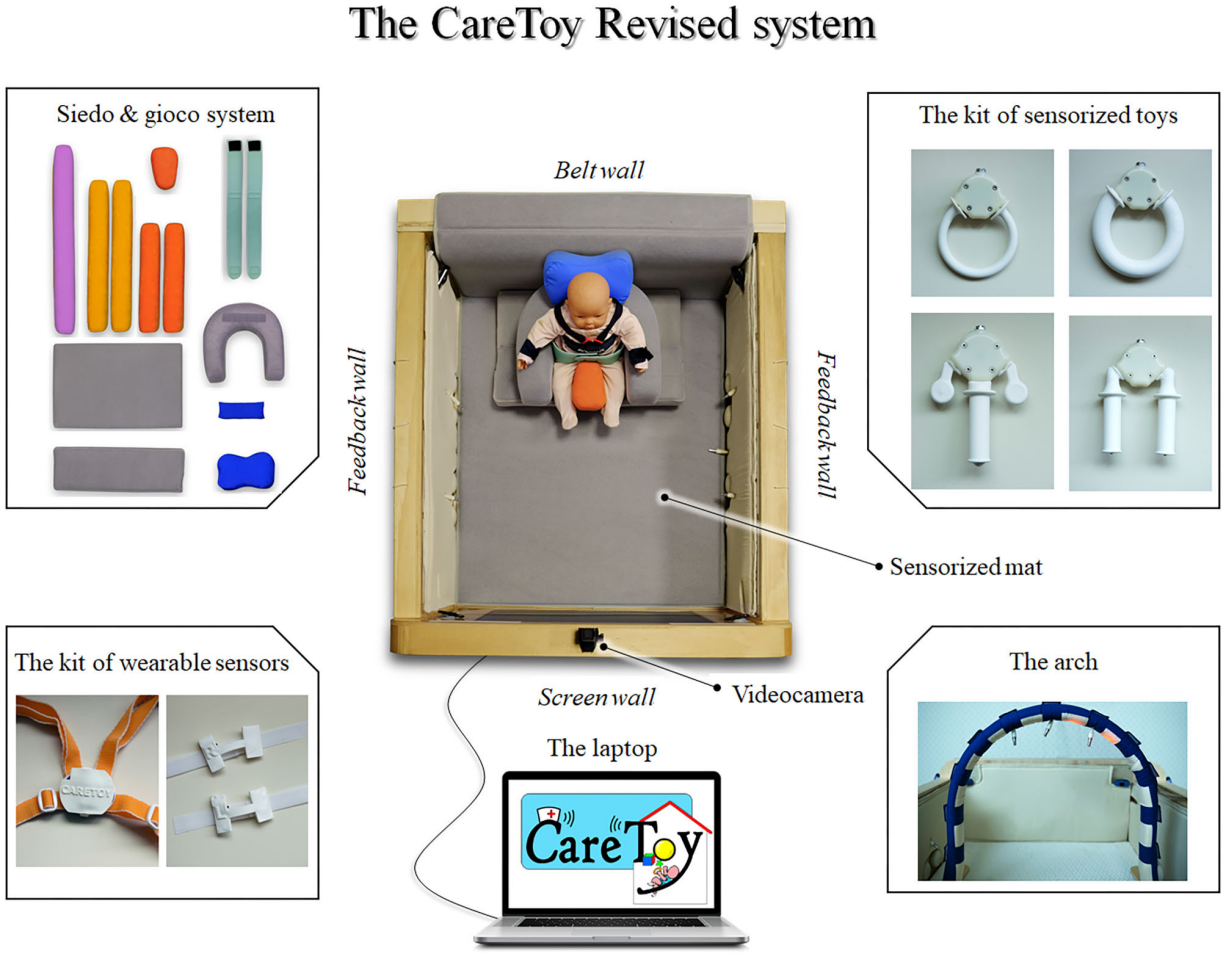
The CareToy-R system.

First of all, due to the crucial postural needs of these infants, a postural modular system was added in the gym. An *ad-hoc* kit of Velcro-strap pillows was realized, using Siedo & Gioco facilities (Fumagalli, Italy), allowing a safe and comfortable positioning of infants in supine, prone, sitting, or side position, with different facilities (see Figure 1).

Together with this structural modification, the content of the goal-directed activities, called CareToy scenarios, was also changed. In detail, thanks to the possibility to set the audio-visual stimuli, the duration, intensity, and features of lights and sounds presented as stimulations and/or feedback have been modified.

Moreover, the video library was changed, adding some pictures and short movies with high contrasted images and customized features, useful for those infants with visual impairments.

The scenarios library was also modified and the clinical and rehabilitative staff made it more functional for the target population. Then, as in the previous projects, CareToy scenarios were further adapted to the individual developmental needs. Indeed, the training can be designed with high complexity and variability depending on the activated modules and features of lights, sounds, videos, and feedback. Scenarios could be planned to train the infant in different positions (supine, sitting, prone, on side), remotely chosen and periodically updated by rehabilitative staff according to infant's needs and capabilities, to promote the personalized goals.

After the T0 assessment, the CareToy-R system was delivered at home of infants randomized to the CareToy-R group; families who did not have an available internet connection were provided with a portable wi-fi router.

In general, the training was planned for 8 weeks, with daily activity between 30 and 45 min, organized in different scenarios (with different goals) lasting from 2 up to 10 min each. The first days of training were planned based on the baseline assessment; then, therapists from remote periodically updated the training, according on infant behavior and/or progress.

Clinical and rehabilitative staff, mainly composed by child neurologists and pediatric physical therapists, followed infants and their families during the whole training period and planned and monitored the customized goal-directed rehabilitative activities called CareToy scenarios; parents were trained on how to use the system and how to play with their infant during the first days of CareToy-R intervention with face-to-face visits with therapists. During the whole training, the research team remotely monitored infants and performed on-site visits on a weekly basis. Additional assistance, with supplementary visits or video calls managed by clinicians together with the technical assistance supplied by bioengineers when necessary, was arranged as required, in order to provide hardware and software support and any additional advices on how to manage the training.

The CareToy-R project was active also during the COVID-19 pandemic period with three trainings provided during the forced lockdown; in these cases, on-site visits were replaced by tele-visits and regular staff meetings were performed online.

### Outcome Measures

The feasibility of CareToy-R training has been evaluated on several measures.

**Feasibility Criteria**

The criteria, based on relevant recommendations for conducting research on feasibility, have been taken from the literature (27–29). The feasibility measures were grouped on the basis of their focus which could be the training intervention or the procedures and study design.

These criteria have been adapted for the CareToy-R study; in details measures have been established as follows:

Feasibility of intervention:

✓Accessibility: intelligibility of information of the scenarios in terms of preparation (use of pillows, toys, etc.) and execution (how to stimulate infant), showed on parents' interface of the laptop
✓Training compliance: required days for completing the 80% of the planned training (at least 8 weeks, that is, the fixed interval of the training)
✓Technical smoothness: good functioning of the CareToy-R system, defined as the quantity of technical issues and malfunctioning experienced
✓Training motivation: motivation and reported effort in carrying out the training.

Feasibility of study design and procedures:

✓Participation willingness: rate of acceptance of the participation in the study
✓Participation rates: number of dropouts
✓Loss to follow-up: recording of all data from all outcome measures
✓Assessment time scale: required interval for collecting all outcome measures (at least 1 week)
✓Assessment procedures: loss to follow-up rates.

The definition and measurement of CareToy-R feasibility criteria is shown in the Table 1.

**Table 1:** Feasibility criteria.

	**Feasibility criteria**	**Definition**	**Feasibility question**	**Feasibility criterion for success**	**Measurement/recording?**
Feasibility of intervention	Accessibility	Intelligibility of information of the scenarios preparation (pillows, toys, etc.) and execution (how to stimulate infant), showed on CT-R interface	Do participants understand all training objectives and rules?	100% of participants understand all activities of the training	Recording of clarification requests to the therapist, number of error message displayed on the laptop
	Training compliance	Duration of the training (at least 8 weeks, that is, the fixed interval of the training)	Do participants perform all training sessions in at least 8 weeks?	A minimum of 80% training completed in 8 weeks	Report from CareToy Admin, i.e., the date of the first and the last day of training
	Technical smoothness	Functioning of CT-R system, defined as the quantity of issues and malfunctioning	Are there technical issues with the training?	90% of participants will be able to perform their training without technical issues	Number of assistance requests
	Training motivation	Motivation and reported effort in carrying out the training	Are participants motivated to perform training intervention?	Difference between planned training days and executed training days	CareToy admin data about planned and executed training
Feasibility of study design and procedure	Participation willingness	Rate of acceptance of participation in the study	What is the participation rate?	At least 80% of eligible participants agreed to join the project	Caretoy database
	Participation rates	Number of dropouts	Do all eligible participants who agree actually perform the training intervention?	80% of participants who gave consensus participated in the study	Caretoy database
	Loss to follow up	Recording of all data from all outcome measures	Can all data be collected without any problems?	90% of the outcome measures were collected	Caretoy database
	Assessment time scale	Required interval for collecting all outcome measures (at least 1 week)	Can follow-up data be collected within a week after the training period?	Time from end of training period to first follow-up data collection	Recorded data of the beginning and the end of the training (CareToy Admin) and data of assessments
	Assessment procedures	Loss to follow-up rates	Is the loss to follow-up acceptable?	Less than 20% of participants fail to complete outcome measures on all follow-up assessments	Collection of data report by examiners

#### The Questionnaires

Considering the crucial role of parents in CareToy-R intervention, it was necessary to assess their ability to use the system and their perception about system usage and training effectiveness. For this reason, all families were asked to fill in two questionnaires, aimed to understand their familiarity with technology and their opinions about the training.

The first tool is a questionnaire already available in literature, that is, the Information Technology (IT) Familiarity Questionnaire, developed by Geyer (24). It has been used to evaluate the participants' familiarity with Information Technology. It consists of 8 questions in 3-points Likert scale (1=daily use, 2=seldom use, 3=never used), investigating the frequency of use of IT. The total score was the average of the scores of the eight questions. This first brief questionnaire was aimed to understand the familiarity with technology of parent who mainly carried out the training.

The second tool is an *ad-hoc* questionnaire called “CareToy-Revised Questionnaire Parent-Infant Experiences (CRQPE),” developed from the acceptability questionnaire of the first CareToy project [(30); see Supplement 1]. It is organized in 44 questions, divided in five areas: general features of CareToy-R system, changes due to the training, easy to use, infant participation, and time dedicated to the training. It is mainly composed by Likert scale answers in five points (where 1 meant “not at all,” up to 5 which meant “yes”) and there are also some open answers in which parents can express their thoughts. All the items of CRQPE were developed, specifically for CareToy-R project, out of the standard definitions of usability (31–33) and acceptability (34, 35).

The two questionnaires were administered in the post-training assessment (T1).

### Data Collection

The clinical staff remote management of the training was possible thanks to the software CareToy Admin, which allows to plan the training choosing scenarios from the library and/or to modify them for a more suitable use for the single infant. Moreover, CareToy Admin collects all the training sessions and automatically provides a detailed report which includes planned and details of executed scenarios. Clinicians have the possibility to check all data of the modules (e.g., sensorized toys, mat, etc.) and the videos of infant play, for specific analyses, detecting the results and planning further training.

At the end of each training, a Microsoft Excel sheet is created, summarizing planned and executed scenarios together with the duration of each scenario, each session (training day) and of the whole training.

The questionnaires were administered to families immediately after the end of the CareToy-R training period (T1) in a face-to-face interview with the parent who was mainly in charge of the training (or both). This allowed an easier administration, as the interviewer was free to explain the questions when necessary and, above all, it gave the opportunity to parents to explain their opinions.

### Statistical Analysis

Clinical data were analyzed by means of Statistical Package for Social Sciences (SPSS, vers. 20.0). Descriptive analyses were used to show the demographic data of infants and of their mothers and the results of questionnaires, for the different areas and the total. Next, multivariate analyses were carried out to explore the differences of treatment planning (i.e., Mean daily CareToy-R training planned in minutes and Total planned training in hours) and of treatment execution (i.e., Mean daily CareToy-R training executed in minutes and Total executed training in hours) and of the total scores and the different domain scores of the CRQE in relation to the infant's and mother's factors. Specifically, for infants, we considered the age at T0 as covariate and the sex (male/female) and the gestational age (prematurity/at term) as fixed factors. For the mothers, we considered the values of “familiarity with technology” as covariate and the nationality (Italian/Foreign) as fixed factor. Moreover, as exploratory analyses, the Mann-Whitney *U*-test was carried out to compare the hours of total training planned and executed in the infants that did the CareToy-R training during the lockdown period respect to the others.

## Results

### Participants

Among the 20 eligible families randomized to the CareToy arm of the RCT, 19 families agreed to participate to the intervention. All participants included in the CareToy-R intervention investigative arm of the CareToy-R project (total: 19 families) accepted to fill in the two questionnaires.

Fourteen families were Italian while five from foreign countries (2 Polish, 1 Albanian, 1 Peruvian, and 1 Ukrainian). Trainings were carried out in different districts of Tuscan region with a mean distance of 79.8 km from IRCCS Fondazione Stella Maris, ranging from Pisa (15 km, the nearest place from Stella Maris) to Arezzo (165 km, the farthest).

The sample of infants was composed by 11 males and 8 females, with a mean age at the beginning of the study of 5.95 ± 2.13 months (range 3.12–10.78 months).

The trainings were assisted exclusively by mothers and their mean age was 32.95 ± 6.49 years (range 18–41 years). Most mothers were employed at the time of the training and had a high school education.

Demographic characteristics of participants (infants) and mothers are shown in Table 2.

**Table 2:** Sample characteristics.

**ID**	**Gestational Age (weeks)**	**Infant Age at T0 (months)**	**Sex**	**Mother age (years)**	**Mother nationality**
#1	40^+5^	4.34	Female	34	Italian
#2	37^+5^	4.67	Female	41	Polish
#3	26^+0^	10.59	Male	36	Italian
#4	40^+0^	6.48	Male	35	Italian
#5	32^+4^	6.12	Male	41	Italian
#6	40^+0^	7.56	Female	36	Italian
#7	40^+0^	5.75	Female	28	Italian
#8	26^+0^	6.84	Female	35	Italian
#9	36^+3^	4.50	Male	31	Italian
#10	33^+0^	5.00	Female	29	Italian
#11	40^+0^	8.68	Male	37	Italian
#12	37^+0^	4.54	Male	26	Italian
#13	39^+0^	4.96	Female	24	Polish
#14	27^+0^	3.12	Male	27	Albanian
#15	40^+0^	4.27	Male	39	Italian
#16	26^+6^	5.92	Male	39	Italian
#17	29^+0^	10.78	Male	18	Peruvian
#18	40^+0^	4.27	Female	29	Italian
#19	40^+0^	4.64	Male	41	Ukrainian
Group results	35.16 ± 5.68	5.95 ± 2.13	11 males 8 females	32.95 ± 6.49	14 Italian 5 foreign

### Feasibility Outcome

Feasibility criteria were all achieved as follows.

First of all, the Feasibility of Intervention:

✓Accessibility: all participants completely understood instructions presented with software and written on printed manual; there were no further requests of clarification. Furthermore, variables related to mothers, as the nationality or the results of the questionnaire “familiarity with technology” did not impact the dose of executed training (Table 3).
✓Training compliance: the clinical staff, on the basis of each infant's developmental need and personal goals, scheduled all the trainings for a total duration of 33 to 55 days, planning a mean daily training which ranged from 27.90 to 39.27 min (mean 34.37 ± 3.15 min) (Table 4). The period of forced lockdown (#2, #12, and #19) due to the emergency of COVID-19 pandemic, did not change the way to plan and deliver the training, and even if not significantly, the total planned and executed training (hours) was higher in the three cases carried out during COVID-19 (28.29 ± 1.53 and 26.42 ± 2.92, respectively) with respect to the others (24.47 ± 4.69 and 21.60 ± 5.28, respectively).

**Table 3:** Mothers characteristics.

	**Nationality (Italian or foreign country)**			**Questionnaire “familiarity with technology”**		
	***F*-test**	**Df**	***p*-value**	***F*-test**	**df**	***p*-value**
Mean daily CT-R training planned (minutes)	0.043	1	0.839	0.146	1	0.708
Mean daily CT-R training executed (minutes)	1.230	1	0.284	0.014	1	0.907
Total planned training (hours)	2.032	1	0.173	3.416	1	0.083
Total executed training (hours)	2.890	1	0.108	1.661	1	0.216

**Table 4:** Infants characteristics and training.

	**Age at T0**			**Sex (male and female)**			**Gestational age (preterm or term)**		
	***F*-test**	**df**	***p*-value**	***F*-test**	**df**	***p*-value**	***F*-test**	**df**	***p*-value**
Mean daily CT-R training planned (minutes)	1.402	1	0.256	0.004	1	0.951	0.208	1	0.656
Mean daily CT-R training executed (minutes)	0.318	1	0.582	2.250	1	0.156	0.100	1	0.756
Total planned training (hours)	1.592	1	0.228	0.069	1	0.797	1.217	1	0.289
Total executed training (hours)	1.049	1	0.323	0.637	1	0.438	0.358	1	0.559

Considering different variables as corrected age of infant at the beginning of the training, sex, and gestational age (expressed as preterm or at term), there were no significant differences in the planned number of days of training and in the planned daily mean duration of the training (Table 4).

All participants completed the training with a total duration of 31 to 55 days (mean 43.21 ± 7.67), carrying out a mean daily training which ranged from 21.34 to 35.83 min (mean 30.30 ± 4.42). The mean total duration of the performed training was 22.40 ± 5.15 h (Table 5).

**Table 5:** CT-R training data.

**ID**	**CT-R Training days planned (N^**°**^)**	**CT-R Training days executed (N^**°**^)**	**Training executed (%)**	**Mean daily CT-R training planned (minutes)**	**Mean daily CT-R training executed (minutes)**	**Total planned training (hours)**	**Total executed training (hours)**
#1	42	38	96%	35.72	23.06	25.01	16.14
#2	55	55	100%	32.20	31.35	29.51	28.74
#3	33	33	100%	39.26	35.64	21.59	19.60
#4	48	48	100%	38.44	35.83	30.75	28.66
#5	33	31	98%	33.52	29.84	18.43	16.41
#6	35	34	99%	27.90	21.34	16.28	13.31
#7	51	51	100%	34.62	31.10	29.43	26.43
#8	43	43	100%	37.56	28.60	23.59	20.49
#9	50	50	100%	34.46	34.34	28.72	28.62
#10	46	43	97%	31.65	26.52	24.26	20.33
#11	50	50	100%	36.36	33.73	30.30	28.11
#12	50	50	100%	31.88	27.77	26.57	23.14
#13	44	42	98%	39.27	34.67	29.45	26.00
#14	50	50	100%	33.79	32.30	28.16	26.92
#15	41	39	98%	28.85	22.40	19.72	15.31
#16	35	35	100%	35.99	34.46	20.99	20.10
#17	33	33	100%	35.07	31.97	19.29	17.58
#18	45	43	98%	33.99	29.85	25.49	22.39
#19	53	53	100%	32.59	31.02	28.78	27.40
Group results (mean±SD)	44.05 ± 7.28	43.21 ± 7.67	99% ± 1%	34.37 ± 3.15	30.30 ± 4.42	25.07 ± 4.54	22.40 ± 5.15

All participants completed the 80% of training in 8 weeks; only four participants needed one extra week to complete the training (total duration: 9 weeks) and this was mainly related to holiday periods.

Moreover, when considering the executed amount of training, there were no significant differences related to the variables: age at the beginning of the training, sex, and gestational age.

✓Technical smoothness: the CareToy-R system experienced two kinds of hardware issues: in one case, it was necessary to replace during the training the laptop of the Caretoy system and in 12 families one of the toys. The software presented some technical issues during 3 trainings. Nevertheless, all these issues were fixed with a remote assistance by a dedicated team of engineers; after a quick replacement of the malfunctioning item, all participants could resume the training after a stop of about a half day.
✓Training motivation: 12 subjects executed the 100% of the planned training days and the other seven subjects between 96 and 99% of the planned training days.

Concerning the feasibility of study design and procedures:

✓Participation willingness: only one eligible family did not give the consent in participating to the project, because they have not the required space in their house for positioning the CareToy-R system.
✓Participation rates: No dropouts were reported.
✓Loss to follow-up: it was possible to record all data of all outcome measures and there were no missing data.
✓Assessment time scale: follow-up measurements of all participants were collected within 1 week after the end of the training (range 0–7 days, mean= 4.53 ± 3.31 days). Only three follow-up measurements were collected between 8 and 15 days after training, because of the holiday period (mainly summer holiday and Christmas).
✓Assessment procedures: all participants who started the training intervention completed the post-training assessments.

### The Questionnaire

All 19 families accepted to fill in the questionnaires and the interview was carried out by therapist during the assessment after the training (T1).

Overall, the answers of the CRQPE questionnaire had a total score all above 110 points (corresponding to 73.33%) with a range of 110–138 points and mean total score of 126.74 ± 8.43 points (84.49%).

Regarding the five sections: “Infant participation” had a range of raw scores between 19 and 30 points, “Easy to use” had a range of raw scores from 21 to 27; the section “General features of CareToy-R system” presented a range from 22 to 29 and “Changes due to the training” showed a range from 21 to 30 and “Time dedicated to the training” between 20 and 29.

In the subsections the percentage of mean raw scores resulted in increasing order: 81.05% in “easy to use” (low percentage obtained), 84.74% in “general features of CareToy-R system,” 84.91% in “time dedicated to the training,” 85.26% in “infant participation,” and 86.49% in “changes due to the training” (higher percentage obtained).

**Figure 2:**
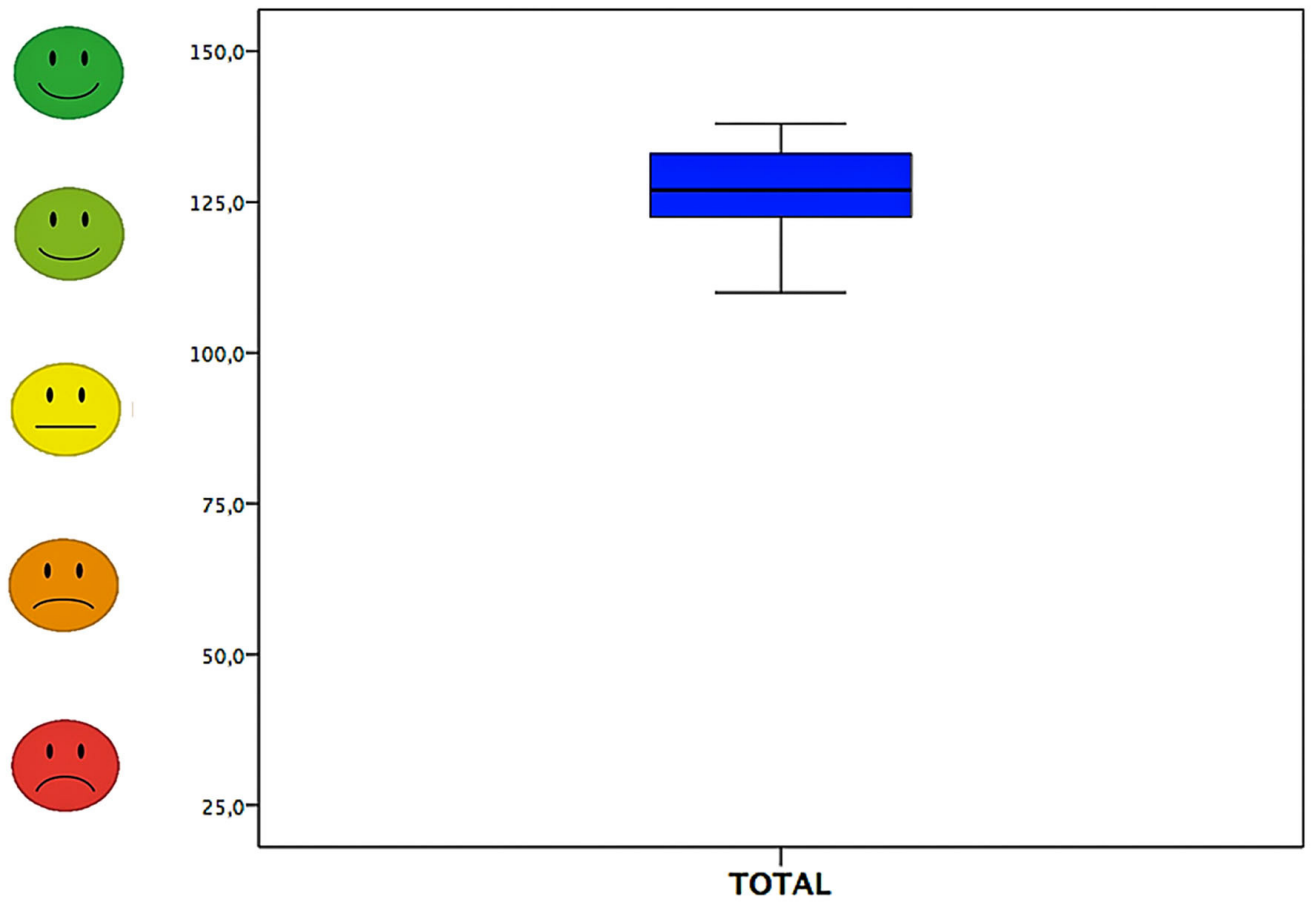
Total answers to the CRQPE.

**Figure 3:**
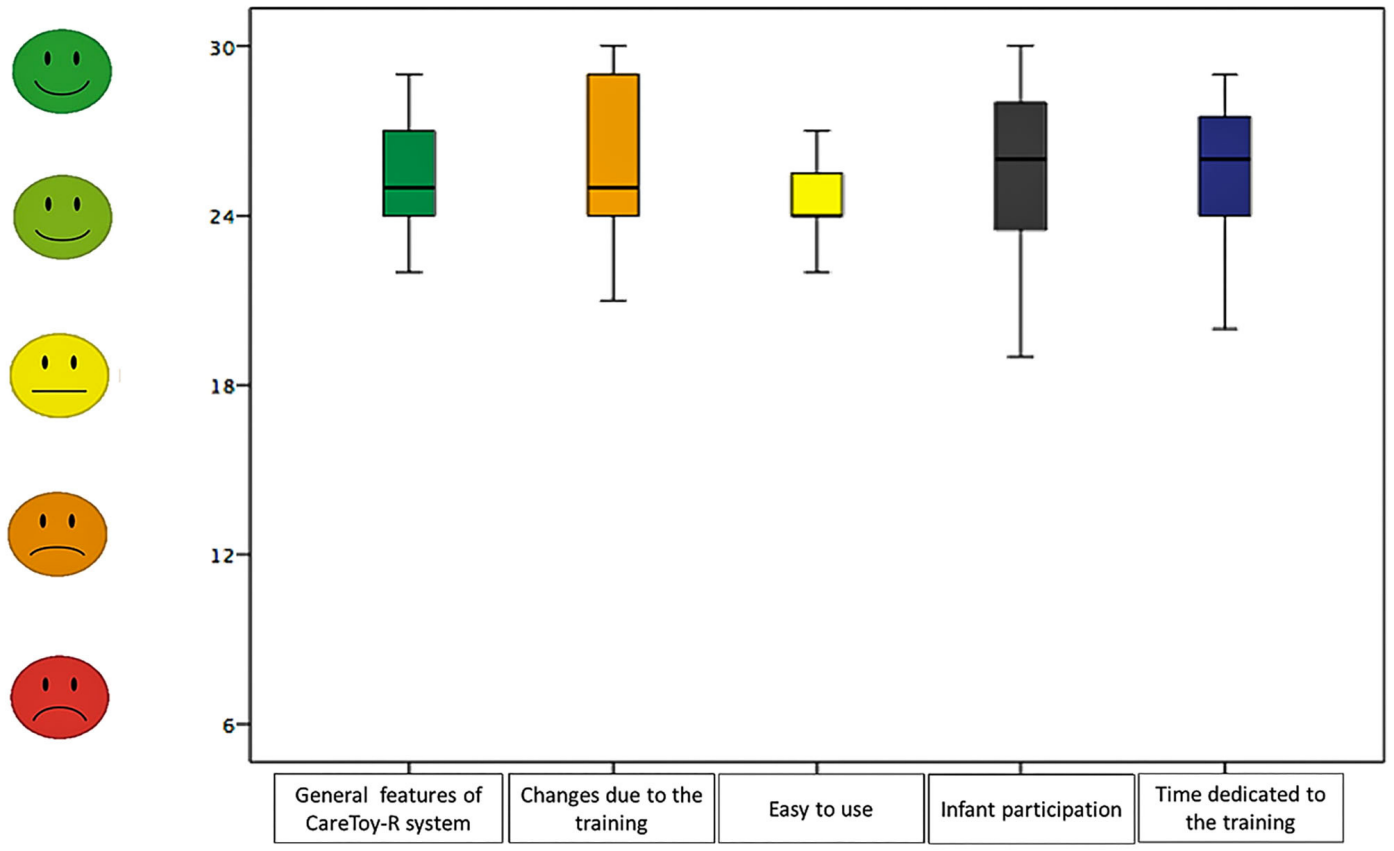
Answers of CRQPE sections.

Median and 95% confidence interval of scores in the questionnaire (both total and section scores) are shown in Table 6 and Figures 2, 3.

**Table 6:** Results of CRQPE questionnaire.

	**Sample**	
	**Median [95% CI]**	
	**Raw score**	**%**
General features of CareToy-R system	25.42 [24.49–26.35]	84.74% [81.65–87.83]
Changes due to the training	25.95 [24.53–27.36]	86.49% [81.78–91.20]
Easy to use	24.32 [23.53–25.10]	81.05% [78.43–83.68]
Infant participation	25.58 [24.19–26.97]	85.26% [80.62–89.91]
Time dedicated to the training	25.47 [24.15–26.79]	84.91% [80.52–89.31]
Total	126.74 [122.67–130.80]	84.49% [81.78–87.20]

Furthermore, the answers of (IT) Familiarity Questionnaire (24) had a raw score range between 8 and 21 points (33.33–87.50%) and mean total raw score of 12 points (51.32%). The total mean presented a range between 1.00 and 2.63 points.

There were no significant effects in the total scores and different sections of CRQPE respect to the sex of the subjects, the age at the beginning of the training, or the gestational age (Table 7).

**Table 7:** Infants' characteristics and CRQPE answer questionnaire.

	**Age at T0**			**Sex (mother and female)**			**Gestational age (preterm or term)**		
	***F*-test**	**Df**	***p*-value**	***F*-test**	**Df**	***p*-value**	***F*-test**	**df**	***p*-value**
General features of CareToy-R system	2.638	1	0.127	0.899	1	0.359	0.039	1	0.901
Changes due to the training	0.921	1	0.354	0.005	1	0.947	0.016	1	0.836
Easy to use	1.058	1	0.321	2.002	1	0.179	0.044	1	0.614
Infant participation	2.012	1	0.178	0.887	1	0.362	0.358	1	0.752
Time dedicated to the training	1.058	1	0.321	0.156	1	0.699	0.104	1	0.683
Total CRQPE	3.233	1	0.094	0.469	1	0.504	0.174	1	0.846

Likewise, there were no differences in the total scores of answers related to mothers' characteristics: nationality and the level of familiarity with technology (Table 8).

**Table 8:** Mothers' characteristics and CRQPE answer questionnaire.

	**Nationality (Italian or foreign country)**			**Results of the questionnaire “familiarity with technology”**		
	***F*-test**	**df**	***p*-value**	***F*-test**	**Df**	***p*-value**
General features of CareToy-R system	0.094	1	0.763	0.45	1	0.835
Changes due to the training	0.056	1	0.816	0.889	1	0.360
Easy to use	0.440	1	0.517	0.498	1	0.490
Infant participation	0.644	1	0.434	0.553	1	0.468
Time dedicated to the training	1.213	1	0.287	0.228	1	0.639
Total CRQPE	0.350	1	0.562	0.011	1	0.918

## Discussion

The present study is the first one in the literature that investigates the acceptability, usability, and feasibility of CareToy-R EI in families with infants at high-risk for CP, evidencing the first milestone in the field of the use of new medical devices in tele-rehabilitation. It is crucial that a new device that is addressed to the home use is feasible in its use because even if it is effective but not feasible, it is hard to be used and translated in the clinical practice. With the current study, we have shown for the first time the applicability of the CareToy-R system and its relevance for home-based early intervention programs in high-risk infants.

For our purpose we referred to the literature for the indication about the methods to assess the usability and acceptability of technologies for home-based rehabilitation (36) and the criteria based on relevant recommendations for conducting research on feasibility, already used in studies (27–29, 37).

The data of this feasibility study highlight different achievements and are in line which the principle of high customized training of the CareToy concept.

Since the first CareToy has been created, many studies have been dedicated to test its effects on preterm infants (9) and its feasibility in other populations (11); the current study presents the first results concerning its feasibility in another delicate population, namely, infants with perinatal brain injury. As compared to the previous CareToy studies, this project not only involves a new and critical population but also introduces some change in the CareToy system and the extension up to 8 weeks of the training duration.

The high rate of acceptance of the CareToy-R project is the first interesting data, which means that the proposal has been agreed upon and the majority of families share this approach and trust the clinical staff in the importance of this EI approach. In one case, a family refused to participate in the project for the lack of enough room to install the CareToy in their house; this could represent a limit to overcome. The scale in which the CareToy-R system was realized was a compromise between the need of including many different sensors and technology and the need to guarantee enough space for the infant move freely; in a future perspective, the use of smaller sensors and hardware components could reduce the size of CareToy, making it more suitable also for families who live in small apartments.

The use of tele-rehabilitation, together with the already known advantages of overcoming the limit of distances and maintaining patients in close contact with their therapists (38), allowed to guarantee the prosecution of the rehabilitation intervention also during the lockdown due to the COVID-19 pandemic emergency, without reducing the dose of the proposed training. The tele-rehabilitation architecture has recently gained much attention due to the possibility of delivering rehabilitative intervention also in periods in which access to healthcare facilities is limited, such as during the sanitary lockdown.

The results of this feasibility study on CareToy-R training confirmed the good functioning of the tele-rehabilitation architecture that in this specific project did not present the typical limit of the unstable or malfunctioning connection (39), thanks to the possibility of delivering a portable router which supplied internet access to those who did not have a personal one or whose connection did not have the required speed. Indeed, connectivity barriers often influence the experience of tele-rehabilitation of patients and clinical staff (40).

Within this project, only families who lived in Tuscany participated in the CareToy-R intervention, and this was due to the recruitment, carried out in the three Neonatology Units of the main Hospitals of this region. An interesting perspective could be to create a more national network, including several Neonatology Units, in order to offer the possibility to join the CareToy-R project also to infants who live in other regions, with the aim of standardizing the methodology and giving the same opportunity of a home-based EI also to infants who live far away from the main clinical centers.

Another first index of CareToy-R feasibility was represented by the clinical and rehabilitative staff management and planning of CareToy-R training and, in particular, by the possibility to program an 8-week training with a daily session of a minimum of 30 min. All the planned scenarios were addressed to meet each specific rehabilitation need; the absence of differences in the amount of planned training among infants who presented different clinical pictures confirms the appropriateness and the high personalization of CareToy-R scenarios.

On their side, families were very compliant also in performing the training. The familiarity of technology, generally high for our sample, seemed not to impact the amount of executed training; this means that the CareToy-R system has been shown as an easy-to-use platform, and the experience in using technological devices does not play a role in the usability of the system.

The high quantity of executed scenarios, not influenced even by the infants' characteristics (sex, gestational age and age at T0) further support the high customization and the focus on each single developmental need and goal of each participant.

As we know, the personalization is of critical importance and the underlying heterogeneity of many disease processes suggests that the strategies for treating an individual with a disease, and possibly monitoring or preventing that disease, must be tailored to match that individual's unique biochemical, physiological, and behavioral profile. This precision medicine, which is one of the new challenges of the research, is not a whole new approach, but it can represent an enhancement of an already used concept aimed to identify, assess, organize and analyze multiple variables to generate a precise and tailored approach. This is not an end-point process, but it includes a number of feedback loops which need ongoing efforts to become ever more precise. Patient data are used to develop clinically relevant models, and the results of these analyze then address the further assessment of patients, as an example of precision medicine as an evolving result (41–44).

In this framework, the CareToy approach showed to have a modular concept and it allows further upgrade for becoming a model of precision medicine, by adding specific and detailed quantitative measurements for each infant allowing a personalized functional profile of infants with different and specific needs.

It is also interesting to consider the dose of performed training together with users' satisfaction in using the system, because they are crucial factors to increase motivation and compliance. On the basis of the CRQPE results, the whole sample of 19 families willingly accepted to fill in the questionnaires and gladly accepted the interview, showing a very good level of acceptability and usability.

CRQPE has demonstrated the possibility of systematically and quantified parental opinions on different features of the CareToy project and the CareToy-R system.

The section “infant participation” had the lowest score, but the score meant that the involvement of the infant in CareToy scenarios was high; together with the high result of the section “changes due to the training” and the data about the amount of training, these data could confirm the suitability of the planned activities to each infant. The areas of the CRQPE relative to the “easy to use” and “general features of CareToy-R system” could be linked to the appreciation of the CareToy-R by families, which had experienced a simple use of the system and liked its features, both in terms of functionality and appearance. Furthermore, despite the variability of expertise in the use of IT, all families reported easy use and returned positive feedback about the feasibility of CareToy-R training, and this further confirmed the usability and acceptability of the CareToy-R system. Finally, the area “time dedicated to the training,” which included questions related to the role of the parent while performing the training, had the highest score of all areas. This means that mothers (who mainly executed CareToy-R training) felt free and confident using the system; moreover, many of them reported that they thought to be empowered after the training. In this sense, the remote guide and support by an expert clinical staff seemed to yield benefits to the parental role.

Looking at the additional comments, the CareToy-R system showed to be, from the parental perception, a useful and innovative tool to promote the development of infants at high neurological risk. The CareToy-R system was indeed widely appreciated in several features by parents, who considered it useful in enhancing and promoting specific skills of their infants on the basis of their individual profile. Parents felt themselves totally involved in their infant's rehabilitation project and they shared activities and playful sequences that allowed them to discover their infant's abilities and potentialities.

Thanks to families' opinions raised from the CRQPE, the CareToy can be improved and optimized for different populations and it could be further be empowered in terms of acceptability and usability. This should support the motivation, encourage parents to perform the training with their infants and, as a consequence, maximize the efficacy of the intervention.

The results of the questionnaires have given an interesting insight directly from the families who participated to the project; this has been useful in order to get feedback on the adequacy of the system and on the appropriateness of the rehabilitation proposals. This vision will serve as an input in order to further improve the CareToy system, correcting those features which are considered less acceptable.

Out of the importance of this feasibility study, some limitations need to be underlined. First of all, concerning the study design, the participation of families in a RCT study and the previous positive results of the CareToy approach in low risk infants can represent a bias because their expectations could affect the answers in the questionnaire. Another bias could be related to the administration way of the CRQPE questionnaire that was carried out by the assessor therapist, blind to the intervention in terms of duration and progressions, as an interview. We chose this way to administer interviews to make parents more confident in talking about their experiences and adding personal comments to the Likert answers, but a positive bias could be raised. Moreover, we have investigated only the parents' point of view but there is the lack in having the feedback of all other end-users opinions than parents, e.g., it would be interesting to create a tool respecting the standard definition of usability and acceptability, aimed to collect data about the point of view of the rehabilitation staff. Moreover, it has not foreseen a detailed cost analysis in order to assess a cost-effectiveness analysis, crucial for estimating the real possibility that CareToy training could have a contribution to reduce the costs of health services and could become relatively inexpensive and can expand the accessibility of rehabilitation to infants at high risk for CP.

Besides these limitations, the current study constitutes the basis of the feasibility of the CareToy-R intervention in high-risk infants, and results of the RCT will show the efficacy of this approach in improving the outcome of this population.

## Data Availability Statement

The raw data supporting the conclusions of this article will be made available by the authors, without undue reservation.

## Ethics Statement

The studies involving human participants were reviewed and approved by Tuscany Pediatric Ethics Committee. Written informed consent to participate in this study was provided by the participants' legal guardian/next of kin.

## Author Contributions

GS and GC conceived the idea for this original research and all other authors contributed to the conception and the design of the study. AC, MC, MG, GS, GC, and RR carried out the enrollment of all infants for the study and their baseline neurological assessment and eligibility. EB, VM, and GS designed and realized the questionnaire. VM performed all the motor assessments and carried out the questionnaires. GS performed the statistical analysis. EB, GS, VM, GC, and RR conceived and prepared the manuscript. All the authors read, critically revised, and approved the final manuscript.

## Conflict of Interest

The authors declare that the research was conducted in the absence of any commercial or financial relationships that could be construed as a potential conflict of interest.

## Caretoy-R Consortium

The CareToy-R Consortium, in addition to the authors of the study, is composed by:

Claudia Artese^1^, Veronica Barzacchi^2^, Marta Cervo^3^, Carlo Dani^1^, Paolo Dario^4^, Marco Di Galante^5^, Ugo Faraguna^6,7^, Patrizio Fiorini^8^, Viola Fortini^3^, Simona Giustini^1^, Clara Lunardi^1^, Irene Mannari^4^, Giada Martini^2^, Martina Maselli^4^, Letizia Padrini^8^ and Filomena Paternoster^8^

^1^ Division of Neonatology, Careggi University Hospital of Florence, Florence, Italy

^2^ Department of Developmental Neuroscience, Istituto di Ricovero e Cura a Carattere Scientifico (IRCCS) Fondazione Stella Maris, Pisa, Italy

^3^ Rehabilitation Department, Children's Hospital A. Meyer, Florence, Italy

^4^ The BioRobotics Institute, Scuola Superiore Sant'Anna, Pisa, Italy

^5^ Istituto di Ricovero e Cura a Carattere Scientifico (IRCCS) Fondazione Stella Maris, Pisa, Italy

^6^ Istituto di Ricovero e Cura a Carattere Scientifico (IRCCS) Fondazione Stella Maris, Calambrone, Pisa, Italy

^7^ Department of Translational Research and of New Surgical and Medical Technologies, University of Pisa, Italy

^8^ Neonatal Intensive Care Unit, Children's Hospital A. Meyer, Florence, Italy
