# Supplementary material for: Feasibility Analysis of CareToy-Revised Early Intervention in Infants at High Risk for Cerebral Palsy
Source: Front Neurol. 2020 Dec 16;11:601137. doi: 10.3389/fneur.2020.601137 (PMC7772394; doi:10.3389/fneur.2020.601137)

“CareToy-Revised Questionnaire Parent-Infant Experiences’

The questionnaire evaluates the experience of tele-rehabilitation with CareToy-R system assessing different areas in detail

GENERAL FEATURES OF CARETOY-R SYSTEM

How much did you enjoy using CareToy-R?

	1	2	3	4	5	
not at all	○	○	○	○	○	very much

Do you think that CareToy-R needs to be changed?

	1	2	3	4	5	
yes, totally	○	○	○	○	○	not at all

Do you find that CareToy-R is safe for your infant?

	1	2	3	4	5	
not al all	○	○	○	○	○	very much

During the training you used the Fumagalli “Siedo & Gioco” system: how many modules were needed to provide postural assistance for your infant?

	1	2	3	4	5	
all of them	○	○	○	○	○	no one

During the training, did you reduce the number of “Siedo & Gioco” modules?

	1	2	3	4	5	
no one	○	○	○	○	○	all of them

Overall, how do you consider the tele-rehabilitation experience?

	1	2	3	4	5	
awful	○	○	○	○	○	excellent

IRCCS FONDAZIONE STELLA MARIS

CareToy

CHANGES DUE TO THE TRAINING

In your opinion, how much has your infant changed after the training?

	1	2	3	4	5	
not at all	○	○	○	○	○	very much

How much has your infant changed his/her postures during the day (supine, prone, on one side, etc..) after the training?

	1	2	3	4	5	
not at all	○	○	○	○	○	very much

In your opinion, how much the attention and/or production of sounds/vocalizations has changed in your infant after the training?

	1	2	3	4	5	
not at all	○	○	○	○	○	very much

In your opinion, how much has the global motor activity changed after the training?

	1	2	3	4	5	
not at all	○	○	○	○	○	very much

In your opinion, how much has manipulation changed after the training?

	1	2	3	4	5	
not at all	○	○	○	○	○	very much

Did you perceive a rehabilitation project in the activities of the training?

	1	2	3	4	5	
not at all	○	○	○	○	○	very much

IRCCS FONDAZIONE STELLA MARIS

CareToy

EASY TO USE

Overall, how easy was the use of the CareToy-R?

	1	2	3	4	5	
challenging	○	○	○	○	○	very easy

How frequently did you experience technical issues with CareToy-R?

	1	2	3	4	5	
all the time	○	○	○	○	○	never

How often did you need technical assistance?

	1	2	3	4	5	
all the time	○	○	○	○	○	never

During the activities, how difficult was to place the modules of Fumagalli "Siedo&gioco" system inside the CareToy-R?

	1	2	3	4	5	
very much	○	○	○	○	○	not at all

How do you judge the instructions for the positioning of the " Siedo&Gioco" system?

	1	2	3	4	5	
hard to understand	○	○	○	○	○	easy to understand

How often did you need therapist's assistance to perform the training?

	1	2	3	4	5	
never	○	○	○	○	○	all the time

IRCCS FONDAZIONE STELLA MARIS

CareToy

INFANT PARTICIPATION

Was your infant comfortable inside CareToy-R?

	1	2	3	4	5	
not at all	○	○	○	○	○	very much

Was your infant engaged during the scenarios?

	1	2	3	4	5	
not at all	○	○	○	○	○	very much

Do you think that scenarios were suitable to the needs of your infant?

	1	2	3	4	5	
not at all	○	○	○	○	○	very much

How much did your infant enjoy the videos?

	1	2	3	4	5	
not at all	○	○	○	○	○	very much

How much did your infant enjoy the toys on the arch?

	1	2	3	4	5	
not at all	○	○	○	○	○	very much

How much did your infant enjoy the lights on the walls?

	1	2	3	4	5	
not at all	○	○	○	○	○	very much

IRCCS FONDAZIONE STELLA MARIS

CareToy

TIME DEDICATED TO THE TRAINING

Did your infant complete the training activities?

	1	2	3	4	5	
no one	○	○	○	○	○	all of them

How do you value the time dedicated to the training?

	1	2	3	4	5	
exaggerated	○	○	○	○	○	appropriate

How much did you feel free to play with your infant during the training?

	1	2	3	4	5	
not at all	○	○	○	○	○	very much

During/after the training, did you change the home environment to meet your infant's needs?

	1	2	3	4	5	
not at all	○	○	○	○	○	very much

Did you change your way of playing with your infant?

	1	2	3	4	5	
not at all	○	○	○	○	○	very much

Would you recommend the CareToy-R project to other families?

	1	2	3	4	5	
not at all	○	○	○	○	○	very much

THANKS FOR YOUR COOPERATION!

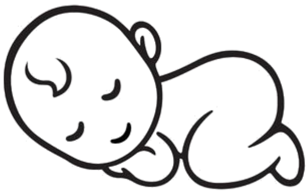

Supplement: Supplementary file 1 [file presentation_1.pdf]
